# A meta-analysis of the epidemiology of giant cell arteritis across time and space

**DOI:** 10.1186/s13075-021-02450-w

**Published:** 2021-03-11

**Authors:** Katherine J. Li, Daniel Semenov, Matthew Turk, Janet Pope

**Affiliations:** 1 Schulich School of Medicine, University of Western Ontario, St. Joseph’s Health Care, 268 Grosvenor St, D2 Rheumatology, London, ON N6A 4V2 Canada; 2 University College Dublin, School of Medicine and Medical Sciences, Dublin 4, Ireland

**Keywords:** GCA, Giant cell arteritis, Temporal arteritis, Meta-analysis, Epidemiology, Geographic variation, Temporal trend, Mortality, Prevalence, Incidence

## Abstract

**Introduction:**

Giant cell arteritis (GCA) is a common large vessel vasculitis in those over age 50 years. This meta-analysis examined the geographical and temporal distribution of the incidence, prevalence, and mortality of GCA.

**Methods:**

A systematic review was conducted using EMBASE, Scopus, and PubMed from their inceptions until 2019. Studies were included if they reported at least 50 or more GCA patients and defined the location and time frame. Articles on mortality were included and standardized mortality ratio (SMR) was extracted where possible. Mean pooled prevalence, incidence, and SMR were calculated using a random effects model. Linear regression was used to explore correlations between latitude and incidence, prevalence, and mortality.

**Results:**

Of the 3569 citations identified, 107 were included. The pooled incidence of GCA was 10.00 [9.22, 10.78] cases per 100,000 people over 50 years old. This incidence was highest in Scandinavia 21.57 [18.90, 24.23], followed by North and South America 10.89 [8.78, 13.00], Europe 7.26 [6.05, 8.47], and Oceania 7.85 [− 1.48, 17.19]. Pooled prevalence was 51.74 [42.04, 61.43] cases per 100,000 people over age 50. Annual mortality was 20.44 [17.84, 23.03] deaths/1000. Mortality generally decreased over the years of publication (*p* = 0.0008). Latitude correlated significantly with incidence (*p* = 0.0011), but not with prevalence, or mortality.

**Conclusions:**

GCA incidence varies nearly 3-fold between regions and is highest in Scandinavia but not significantly. Mortality may be improving over time.

**Supplementary Information:**

The online version contains supplementary material available at 10.1186/s13075-021-02450-w.

## Key messages

- Certain regions have a disproportionate burden of giant cell arteritis (GCA), and the mechanism is not fully understood.
- Latitude influences the distribution of some autoimmune conditions, however, not GCA.
- Despite increasing average age of GCA, increasing GCA rates were not found.

## Introduction

Giant cell arteritis (GCA) is a polygenic immune-mediated disease of unknown etiology that occurs in individuals aged 50 years and older [1]. It is thought to be caused by exaggerated immune responses to vascular endothelial injury with lymphocyte proliferation and giant cell formation. These responses lead to luminal narrowing and disruption of the internal elastic lamina, which limit blood flow and cause end organ ischemia [2]. Common symptoms include headache, scalp tenderness, jaw claudication, and visual loss. GCA is classified using the 1990 ACR criteria, and though a histological diagnosis is preferred, a positive temporal artery biopsy (TAB) is not mandated [3]. Diagnoses may be confirmed without a positive biopsy and imaging is sometimes used such as temporal artery ultrasound.

GCA is a common systemic vasculitis in adults and is closely associated with polymyalgia rheumatica (PMR); approximately 40–60% of patients with GCA have PMR while 15–20% of those with PMR develop GCA symptoms [4].

The epidemiology of GCA has been extensively studied. The average age of diagnosis has increased from 75 in the 1950s to 79 in the 2000s [5]. GCA has also more common in females at a ratio of 2.5:1 [1, 6]. Incidence has consistently been found to be highest in Scandinavia and lowest in Asian countries [1]. Some autoimmune disorders such as multiple sclerosis have shown latitude-associated trends [7]. GCA may be increased in northern latitudes. Seasonal and temporal clustering of incident GCA have been reported, perhaps due to viral triggers; however, this relationship remains unclear [1].

As the population continues to age, the incidence, prevalence, and mortality of GCA are expected to increase. Considering the significant morbidity associated with GCA from blindness, aortic defects, and treatment, a better understanding of the changing epidemiology is needed.

The last major epidemiological meta-analysis of GCA was published in 2008 [8]. Our study aimed to provide a comprehensive update on the global geographic and temporal trends for incidence, prevalence, and mortality in GCA, and examine its potential connection with latitude.

## Methods

### Study selection

A systematic review of the literature was performed to identify studies examining the incidence, prevalence, or mortality of GCA. EMBASE, Scopus, and PubMed were searched from their inceptions until February 2019. Our search strategy is reported in Supplementary Table 1. Studies were included if they were written in English, presented a cohort or cross-sectional data on more than 50 patients with GCA and reported on population, location and/or time-frame parameters. Articles on mortality were included as a rate, and if they provided an age- and gender-matched population (Standardized Mortality Ratio), that was also extracted. Review articles, case-control studies, and case series were excluded. Study quality was assessed by using the Strengthening the Reporting of Observational Studies in Epidemiology (STROBE) checklist (Supplementary Table 2).

### Data extraction and analysis

The following data were extracted from each study: year of publication, study country, total number of GCA cases, years of cohort, number of deaths, incidence, prevalence, and mortality rate. Mortality rate was standardized across cohorts to deaths per 1000 people per year. The website www.latlong.net was used to determine the latitude of the population location (city or region) examined in each included article. Forest plots were generated using Revman5.3 to determine the pooled incidence, prevalence, and mortality using Wilson’s score method. The 95% confidence intervals were generated using a random effects model to account for differences in variance and quality between studies. Tau squared statistics were used to evaluate heterogeneity between studies. Publication bias was assessed using funnel plots, which were also generated on Revman5.3. Linear regression was used to evaluate temporal and geographic differences using SPSS26 where *p* < 0.05 was significant.

## Results

### Search results

**Fig. 1:**
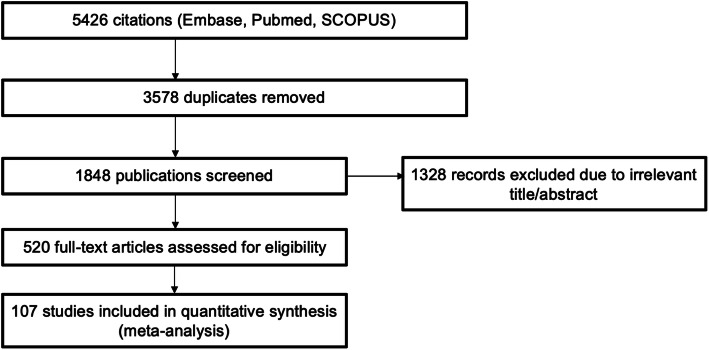
Flow chart of study selection. The flow chart was developed using PRISMA: Preferred Reporting Items for Systematic Reviews and Meta-Analyses

The search identified 5426 articles of which 107 were included for analysis (Fig. 1). There were 3578 duplicates between databases and 1741 did not meet eligibility criteria. Table 1 provides the information on the studies extracted and the incidence, prevalence, and mortality from each paper. The study quality as measured by the STROBE instrument is found in Table 1. There was some evidence of publication bias according to funnel plots (Supplementary Fig. 1).

**Table 1:** Characteristics and research quality of studies reporting the incidence (average per 100,000 over 50 years old), prevalence (average per 100,000 over 50 years old) and mortality (average per 1000 people over 50 years of age) of GCA

Epidemiological study category	First author (citation)	Year	Location	Latitude (°N)	STROBE	Total patients	Rate
Mortality	Andersson R	1986	Sweden	57.71	20	90	29.17
Mortality	Baslund B	2014	Denmark	55.33	21	1787	26.3
Mortality	Belvedere LM	2016	Italy	49.28	–	280	21.84
Mortality	Bengtsson BA	1981	Sweden	57.70	17	90	28.89
Mortality	Bisgard C	1991	Denmark	56.36	18	34	40.72
Mortality	Boesen P	1987	Denmark	55.33	18	46	27.17
Mortality	Brekke LK	2015	Norway	60.39	–	820	13.51
Mortality	Brekke LK	2016	Norway	60.39	–	820	13.81
Mortality	Catanoso M	2014	Italy	44.70	–	285	15.59
Mortality	Catanoso M	2017	Italy	44.70	19	285	16.76
Mortality	Crow R	2009	USA	40.76	21	44	34.09
Mortality	Diamantopoulos AP	2014	Norway	58.14	22	212	18.87
Mortality	Graham E	1981	UK	51.51	18	90	35.56
Mortality	Gran JT	2001	Norway	58.83	20	338	20.41
Mortality	Hachulla E	2001	France	50.63	19	133	17.13
Mortality	Hernandez-Rodriguez J	2002	Spain	41.39	19	75	12.38
Mortality	Huston KA	1978	USA	43.83	21	42	20
Mortality	Khalifa M	2009	Tunisia	35.83	20	96	2.45
Mortality	Knorring J	1979	Finland	60.17	15	53	15.09
Mortality	Kobayashi S	2003	Japan	35.69	21	66	45.45
Mortality	Labarca C	2015	USA	43.16	21	286	16.08
Mortality	Lie JT	1995	USA	38.58	19	72	10
Mortality	Lin L	2018	UK	55.90	22	9778	14.58
Mortality	Macchioni P	2018	Italy	44.70	22	281	7.94
Mortality	Matteson EL	1996	USA	43.83	20	214	19.08
Mortality	Mohammad A	2011	Sweden	55.99	22	792	24.38
Mortality	Mohammad AJ	2015	Sweden	55.99	22	840	25.55
Mortality	Moinet F	2017	Martinique	14.60	–	40	6
Mortality	Ninan J	2011	Australia	− 34.93	20	225	22.54
Mortality	Nordborg E	1989	Sweden	57.70	20	284	28.87
Mortality	Pamuk ON	2009	Turkey	41.68	21	19	35.09
Mortality	Pierluigi M	2016	Italy	44.70	–	280	16.33
Mortality	Pierluigi M	2018	Italy	44.70	21	285	21.84
Mortality	Rajala S	1993	Finland	61.50	19	66	22.73
Mortality	Tam S	2008	Hong Kong	22.40	21	19	52.63
Mortality	Whitfeild AG	1963	UK	52.63	10	72	13.89
Mortality	Yates M	2013	UK	52.63	21	119	21.01
Prevalence	Boesen P	1987	Denmark	55.33	18	46	135
Prevalence	Catanoso M	2017	Italy	44.70	20	285	87.9
Prevalence	Crowson CS	2016	USA	43.83	20	248	204
Prevalence	Herlyn K	2014	Germany	53.87	20	150	44
Prevalence	Khalifa M	2009	Tunisia	35.83	18	96	7
Prevalence	Kobayashi S	2003	Japan	35.69	20	66	1.47
Prevalence	Martinez PJM	2016	Argentina	− 34.60	–	90	120
Prevalence	Pamuk ON	2009	Turkey	41.68	20	19	20
Prevalence	Romero-Gomez C	2015	Spain	36.51	21	29	12.2
Incidence	Abdul-Rahan AM	2011	New Zealand	− 46.47	20	70	12.73
Incidence	Baldursson O	1994	Iceland	64.13	18	133	27
Incidence	Bas-Lando M	2007	Israel	31.77	19	206	11.3
Incidence	Bengtsson BA	1981	Sweden	57.70	18	126	28.6
Incidence	Boesen P	1987	Denmark	55.33	20	46	76.6
Incidence	Brekke LK	2017	Norway	60.39	–	881	16.8
Incidence	Brekke LK	2015	Norway	60.39	–	820	15.7
Incidence	Bustamante ME	2004	Spain	41.39	21	55	4.1
Incidence	Catanoso M	2014	Italy	44.70	21	285	3.3
Incidence	Catanoso M	2017	Italy	44.70	21	285	7.8
Incidence	Chandran AK	2015	USA	43.83	21	74	19.8
Incidence	Dadoniene J	2005	Lithuania	54.69	18	11	0.72
Incidence	Devauchelle-Pensec V	2018	France	45.76	21	241	8.5
Incidence	Diamantopoulos AP	2014	Norway	58.14	–	135	17.2
Incidence	Dunstan E	2014	Australia	− 34.93	–	314	3.2
Incidence	Elfving P	2016	Finland	62.89	20	8	7.5
Incidence	Friedman G	1982	Israel	31.77	20	46	0.49
Incidence	Gonzalez-Gay MA	2003	Spain	43.01	20	210	9.75
Incidence	Gonzalez-Gay MA	1992	Spain	43.01	20	255	6
Incidence	Gonzalez-Gay MA	1997	Spain	43.01	21	93	9.38
Incidence	Gonzalez-Gay MA	1999	Spain	43.01	20	110	14.1
Incidence	Gonzalez-Gay MA	2001	Spain	43.01	20	161	10.24
Incidence	Gonzalez-Gay MA	2007	Spain	43.01	19	255	10.13
Incidence	Gran JT	1997	Norway	58.83	20	66	29
Incidence	Haugeberg G	1998	Norway	58.83	20	42	27.9
Incidence	Haugeberg G	2003	Norway	58.83	18	70	32.4
Incidence	Huston KA	1978	USA	43.83	21	42	11.7
Incidence	Ing EB	2019	Canada	44.23	21	35	4.9
Incidence	Jonasson F	1979	UK	55.90	–	136	4.2
Incidence	Khalifa M	2009	Tunisia	35.83	20	96	7
Incidence	Machado EBV	1988	USA	43.83	19	94	17
Incidence	Mader T	2009	USA	61.22	19	122	1.02
Incidence	Martinez PJM	2016	Argentina	− 34.60	–	90	8.6
Incidence	Mohammad A	2011	Sweden	55.99	–	840	11.3
Incidence	Mohammad AJ	2015	Sweden	55.99	–	840	14.1
Incidence	Mollan SP	2015	UK	52.49	17	7864	4.31
Incidence	Nesher G	2016	Israel	31.77	20	140	8.1
Incidence	Nordborg C	2003	Sweden	57.70	20	665	22.2
Incidence	Nordborg E	1989	Sweden	57.70	18	284	18.3
Incidence	Pamuk ON	2009	Turkey	41.68	21	19	1.13
Incidence	Petri H	2015	UK	55.90	20	4671	11.2
Incidence	Petursdottir V	1999	Sweden	57.70	19	665	22.2
Incidence	Potocnik N	2014	Slovenia	46.06	–	97	8.7
Incidence	Potocnik N	2018	Slovenia	46.06	–	169	10.5
Incidence	Pucelji NP	2018	Slovenia	46.06	21	169	8.7
Incidence	Rajala SA	1993	Finland	61.50	20	66	7.2
Incidence	Ramstead C	2007	Canada	52.13	19	141	9.4
Incidence	Reinhold-Keller E	2000	Germany (north)	54.21	20	180	8.7
Incidence	Reinhold-Keller E	2000	Germany (south)	48.66	20	180	9.4
Incidence	Richier Q	2018	France	44.29	–	60	2.33
Incidence	Romero-Gomez C	2015	Spain	36.51	21	29	2.2
Incidence	Salvarani C	1991	Italy	44.70	20	43	6.9
Incidence	Salvarani C	1995	USA	43.83	18	125	17.8
Incidence	Salvarani C	2004	USA	44.70	20	173	18.8
Incidence	Schmidt D	2001		45.44	20	99	2.07
Incidence	Smeeth L	2006	UK	52.49	20	3928	8.42
Incidence	Smith CA	1983	USA	31.96	18	26	1.58
Incidence	Sonnenblick M	1994	Israel	31.77	20	84	10.2
Incidence	Tam	2008	Hong Kong	22.40	19	47	0.34
Incidence	Udayakumar PD	2013	USA	43.83	–	39	19.25
Incidence	Yates M	2013	UK	52.63	21	119	6.8

Note: Latitude was determined for regions within a country where a study population was taken

### Incidence

**Fig. 2:**
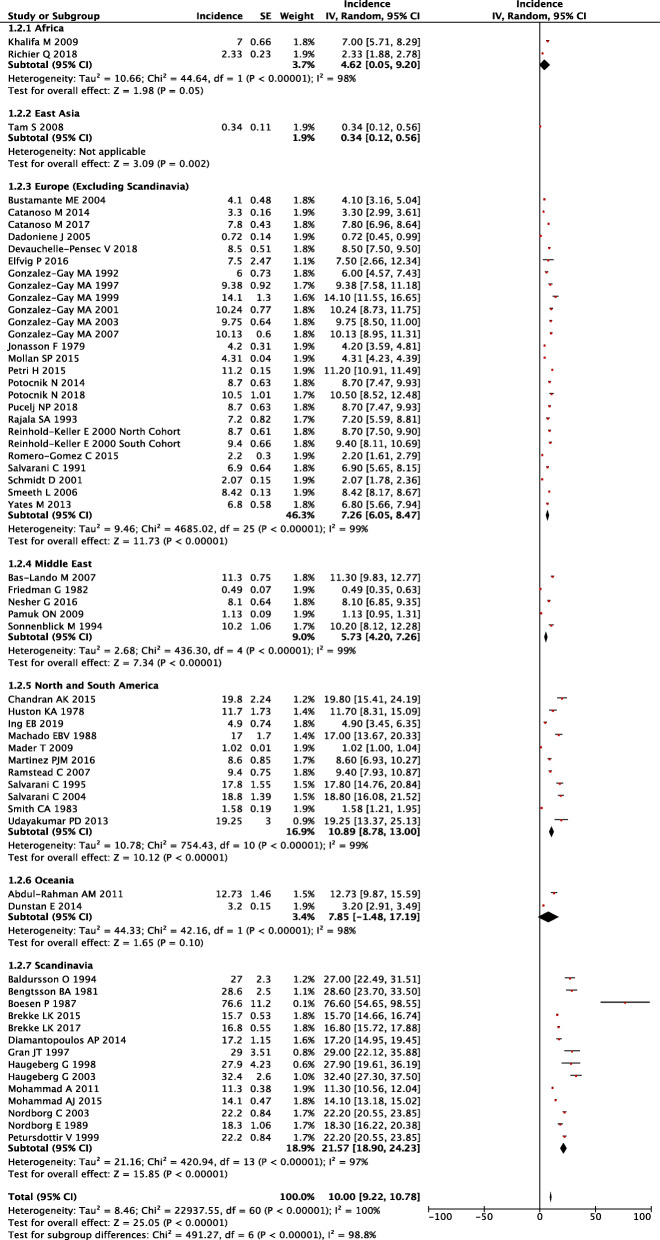
Forest plot of incidence of giant cell arteritis across geographic regions

Of the 107 studies, 61 studies reported on the incidence of GCA. Studies were sorted into several geographic areas and pooled incidence was calculated per 100,000 people over 50 years (Fig. 2). The included geographic areas from highest to lowest incidence [95% CI] were Scandinavia 21.57 [18.90, 24.23], North and South America 10.89 [8.78, 13.00], Oceania 7.85 [1.48, 17.19], Europe 7.26 [6.05, 8.47], Middle East 5.73 [4.20, 7.26], Africa 4.62 [0.05, 9.20], and East Asia 0.34 [0.12, 0.56]. The highest incidence within the studies was in Denmark 76.6 [54.65, 98.55] and the lowest was in Hong Kong 0.34 [0.12, 0.56]. The global pooled incidence was 10 [9.22, 10.78] per 100,000 people over 50 years. Global incidence rates were visually plotted on a map (Supplementary Fig. 2).

Incidence was also assessed across publication years using linear regression. Scandinavia had the largest decreasing incidence rate. Incidence decreased by 0.80 per 100,000 people per year, corresponding to a reduction of two-thirds between 1981 (42.3 per 100,000) and 2017 (13.4 per 100,000) (*R*^2^ = 0.58, *p* = 0.029). Globally, pooled incidence decreased over time at a rate of 0.41 per 100,000 per year (*R*^2^ = 0.27, *p* = 0.034).

### Prevalence

**Fig. 3:**
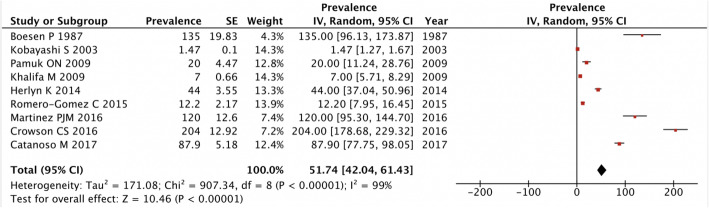
Forest plot of prevalence of giant cell arteritis

A total of 9 studies reported on the prevalence of giant cell arteritis. The overall pooled prevalence was 51.74 [42.04, 61.43] cases per 100,000 people over 50 years (Fig. 3). The prevalence was stable across publication years of the studies using a linear fit model.

### Mortality

**Fig. 4:**
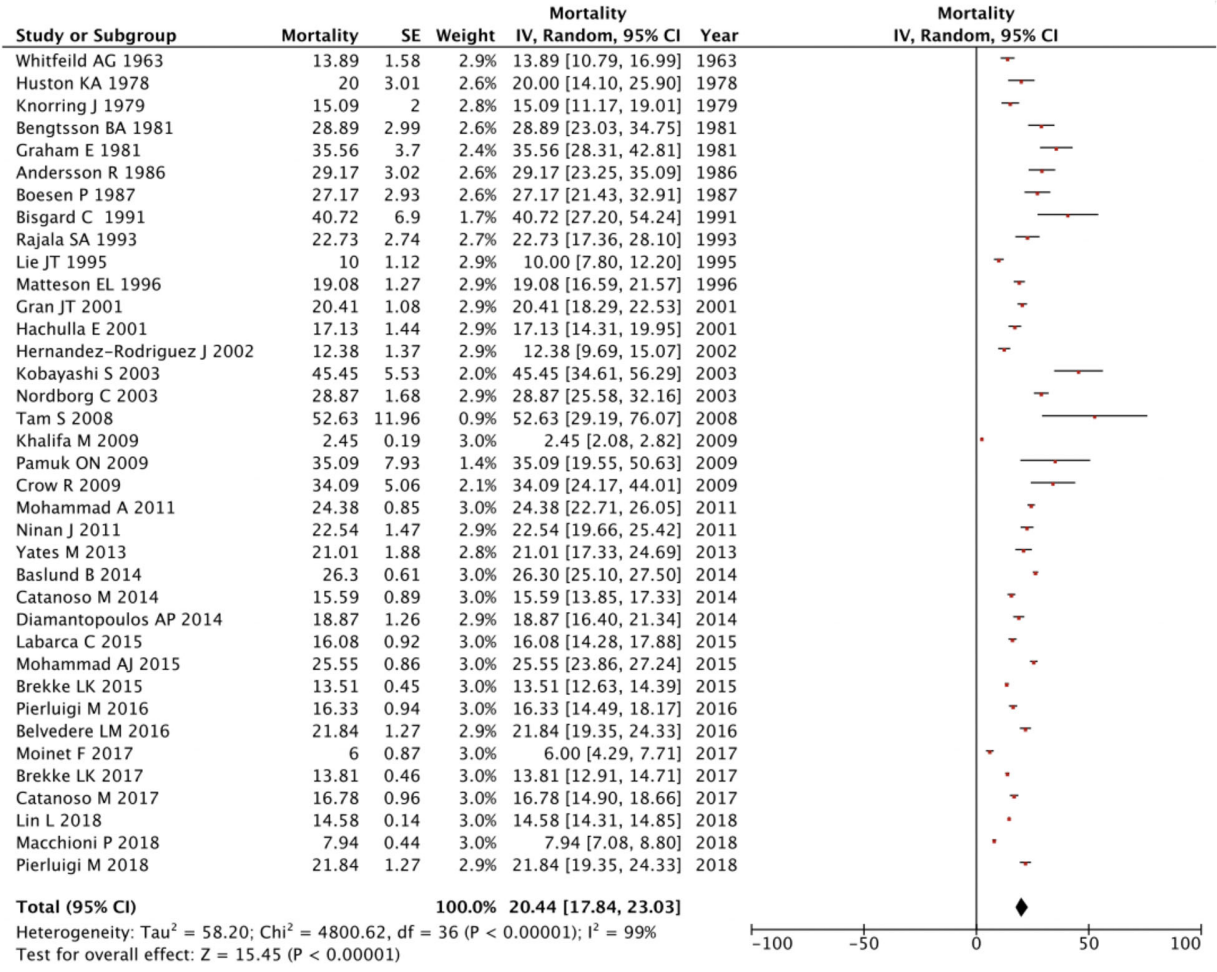
Forest plot of mortality of giant cell arteritis

Thirty-seven articles included data on mortality. The overall pooled mortality rate is 20.44 [17.84, 23.03] cases per 1000 people over 50 years (Fig. 4). Highest mortality was in Hong Kong (52.63) with lowest in the USA (34.09). Across publication years, there was an overall decrease in mortality over time with a rate of 0.14 per 1000 people per year (*p* = 0.00076).

### Latitude

**Fig. 5:**
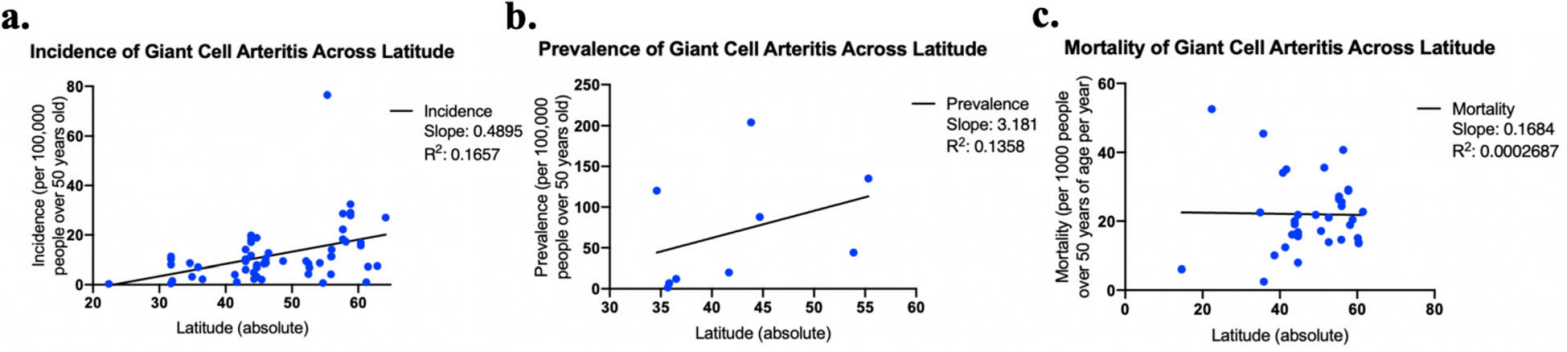
Incidence, prevalence, and mortality of giant cell arteritis across latitude

Incidence, prevalence, and longitude were plotted against absolute latitude and were assessed using linear regression (Fig. 5). The R squared value for incidence, prevalence, and mortality was 0.1657, 0.1358, and 0.0002. Our linear model only showed a significant correlation between latitude and incidence (*p* = 0.0011, beta = 0.489), not prevalence (*p* = 0.33) or mortality (*p* = 0.92).

## Discussion

The incidence of GCA was threefold higher in Scandinavia relative to the rest of Europe and was 6 times higher in Scandinavia compared to East Asia. The high incidence rates of GCA in parts of North America may be explained by communities with Scandinavian ancestry, such as in Olmsted County, USA [9]. This could disproportionately elevate the overall incidence of GCA in North America, with a rate exceeding most regions (except Scandinavia). These findings are consistent with those previously reported [1, 8, 10–13].

The increased incidence of GCA in Scandinavian countries may be explained by genetic susceptibility. Patients with GCA have haplotype variation in certain MHC class II alleles, with a predominance of HLA DRB1*04 specifically. Polymorphisms in genes expressing inflammatory mediators such as TNF, adhesion molecules, and IL18 are sometimes implicated in GCA [1, 14]. In addition, rates could be higher in Scandinavia due to more advanced healthcare tracking systems [15]. Seasonal variations have also been reported, albeit with low statistical power [1]. It is also speculated that microorganisms may trigger infections and lead to immune-mediated hypersensitivity, although evidence for this remains controversial [16].

Our results found that there was only a statistically significant association between latitude and incidence, not prevalence or mortality. However, regional differences may exist due to variations in population concentration; urban populations tend to have higher incidence rates of GCA compared to rural regions [17]. This trend is possibly explained by proximity to medical centers with greater diagnostic capacity and higher surrounding patient concentrations [17]. It is not correct to assume that latitude is associated with increased incidence due to HLA gene concentrations in Scandinavian countries. While Scandinavian countries have both high concentrations of HLADRB1*04 and high latitudes, they are independently associated with the incidence of GCA in both univariate and multivariate models [18]. It is paradoxical that incidence was associated with latitude whereas prevalence was not. The discrepancy between the two likely lies in the statistical power of our prevalence model; as only 9 studies reported on prevalence of GCA compared to 61 that reported on incidence. We expect both incidence and prevalence of GCA would have similar associations with latitude if there were more studies and an analysis of more areas within the world so latitude could be sufficient sufficiently explored for the prevalence in GCA.

With respect to temporal trends, incidence rates globally were found to generally decrease over time, with some regional differences. Specifically, North America, South America, and Europe had stable incidence rates over time, whereas rates in Scandinavia decreased. The downward trend in Scandinavia may be explained by changes in immigration. Immigration to Sweden has been steadily rising since 2008, reaching record high numbers in 2013 [10]. As of 2017, Statistics Sweden reported that around 2,439,007 or 24% of Swedish residents were foreign born [19] Most of these immigrants originated from Asian and Middle Eastern countries, where rates of GCA are among the lowest [20]. Denmark and Norway similarly underwent increases in immigration, albeit with lower numbers [13]. The timeframe over which these increases in immigration occurred coincides with when the decreasing trends in incidence of GCA began (approximately 2011, according to our data). This trend is in sharp contrast with previous epidemiological studies published prior to 2011, which showed increasing incidence over time across Scandinavia and other parts of Europe [9, 11, 12].

We expect total numbers of cases to begin increasing in the future as the population ages.

Mortality in GCA was found to generally decrease over time, and showed no geographic variation. The decrease in mortality can likely be explained by overall increased longevity in the elderly, earlier diagnoses, increased surveillance, and earlier initiation of therapy as well as possibly the use of steroid-sparing treatments such as methotrexate. We had insufficient data to analyze the SMR and cannot comment if the mortality relative to the age-/gender-matched population is changing. Controversy exists surrounding studies with an increased mortality in GCA [1, 11, 21–23].

Our study is not without limitations. There is a lack of a standardized definition of GCA used consistently in the literature, resulting in the inclusion of heterogeneous data in our analysis. This inconsistency is evident in administrative databases, where the lack of a specific billing code for GCA can misclassify patients and either over- or underestimate data [24]. Another consequence is that inclusion criteria were inconsistently used in the study selection process. As previously mentioned, the 1990 ACR criteria do not mandate biopsy positive results. Thus, the majority of hospital-based studies included only biopsy-proven cases, whereas most population or community-based studies included also clinical diagnoses. Therefore, data may be vary depending on which inclusion criteria was used. Non-English studies were also excluded. Finally, some studies on mortality combined data for both PMR and GCA, which would have falsely deflated the reported mortality rates since PMR has a lower mortality rate than GCA [25].

## Conclusion

This study demonstrates epidemiological trends in GCA with a comprehensive description of updated global pooled incidence, prevalence, and mortality of GCA. Incidence rates vary significantly between regions and are highest in Scandinavia. Temporally, GCA incidence and mortality decreased, while prevalence remained stable. Latitude does influence incidence but not prevalence or mortality in GCA although the results may be underpowered for comparing prevalence and latitude in GCA.

## Supplementary Information

**Additional file 1: Supplementary Table 1.** Search terms used for determining the incidence, prevalence and mortality of giant cell arteritis. **Supplementary Figure 1.** Funnel Plots of Incidence, Prevalence and Mortality of Giant Cell Arteritis. **Supplementary Figure 2.** Global Incidence of Giant Cell Arteritis on the World Map.

**Additional file 2: Supplementary Table 2.** STROBE asssessment of the included trials.

## Data Availability

Raw data is presented in Table 1 and Supplementary Table 1. Further information is available upon reasonable request.

## Abbreviations

ACR: American college of rheumatology
GCA: Giant Cell Arteritis
PMR: Polymyalgia rheumatica
SMR: Standardized mortality ratio
STROBE: Strengthening the Reporting of Observational Studies in Epidemiology
TAB: Temporal artery biopsy
