# Supplementary material for: A meta-analysis of the epidemiology of giant cell arteritis across time and space
Source: Arthritis Res Ther. 2021 Mar 11;23:82. doi: 10.1186/s13075-021-02450-w (PMC7948334; doi:10.1186/s13075-021-02450-w)
Supplement: Supplementary file 1 — Additional file 1: Supplementary Table 1. Search terms used for determining the incidence, prevalence and mortality of giant cell arteritis. Supplementary Figure 1. Funnel Plots of Incidence, Prevalence and Mortality of Giant Cell Arteritis. Supplementary Figure 2. Global Incidence of Giant Cell Arteritis on the World Map. [file 13075_2021_2450_MOESM1_ESM.docx]

**Supplementary Table 1** Search terms used for determining the incidence, prevalence and mortality of giant cell arteritis

| GCA (OR) | AND | Epidemiological terms (OR) |
| --- | --- | --- |
| arteriitis,giant cell |  | Incidence |
| arteritis,cranial |  | incidence rate |
| arteritis,giant cell |  | rate,incidence |
| giant cell arteriitis |  | prevalence |
| GCA |  | prevalence study |
| arteriitis temporalis |  | mortality |
| arteritis temporalis |  | excess mortality |
| arteritis,temporal |  | mortality model |
| cranial arteritis |  | death rate |
| headache,histamine |  | death rate model |
| headache,horton |  | fatal outcome rate |
| histamine cephalalgia |  | fatality rate |
| histamine headache |  | lethal outcome rate |
| Horton disease |  | rate,mortality |
| horton headache |  | mortality risk index |
| horton syndrome |  | risk,mortality |
| migrainous neuralgia |  | mortality, premature |
| neuralgia,migrainous |  | survival |
|  |  | disease specific survival |

**Supplementary Figure 1 Funnel Plots of Incidence, Prevalence and Mortality of Giant Cell Arteritis**

**a Incidence, b Prevalence c Mortality**


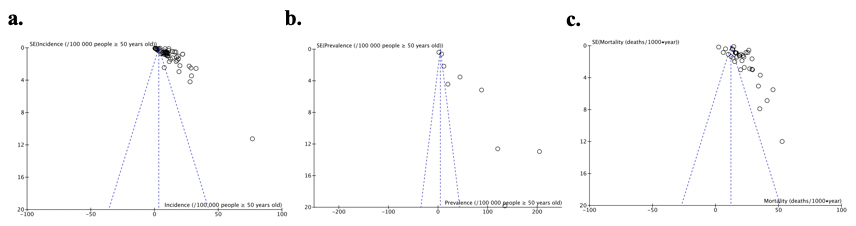


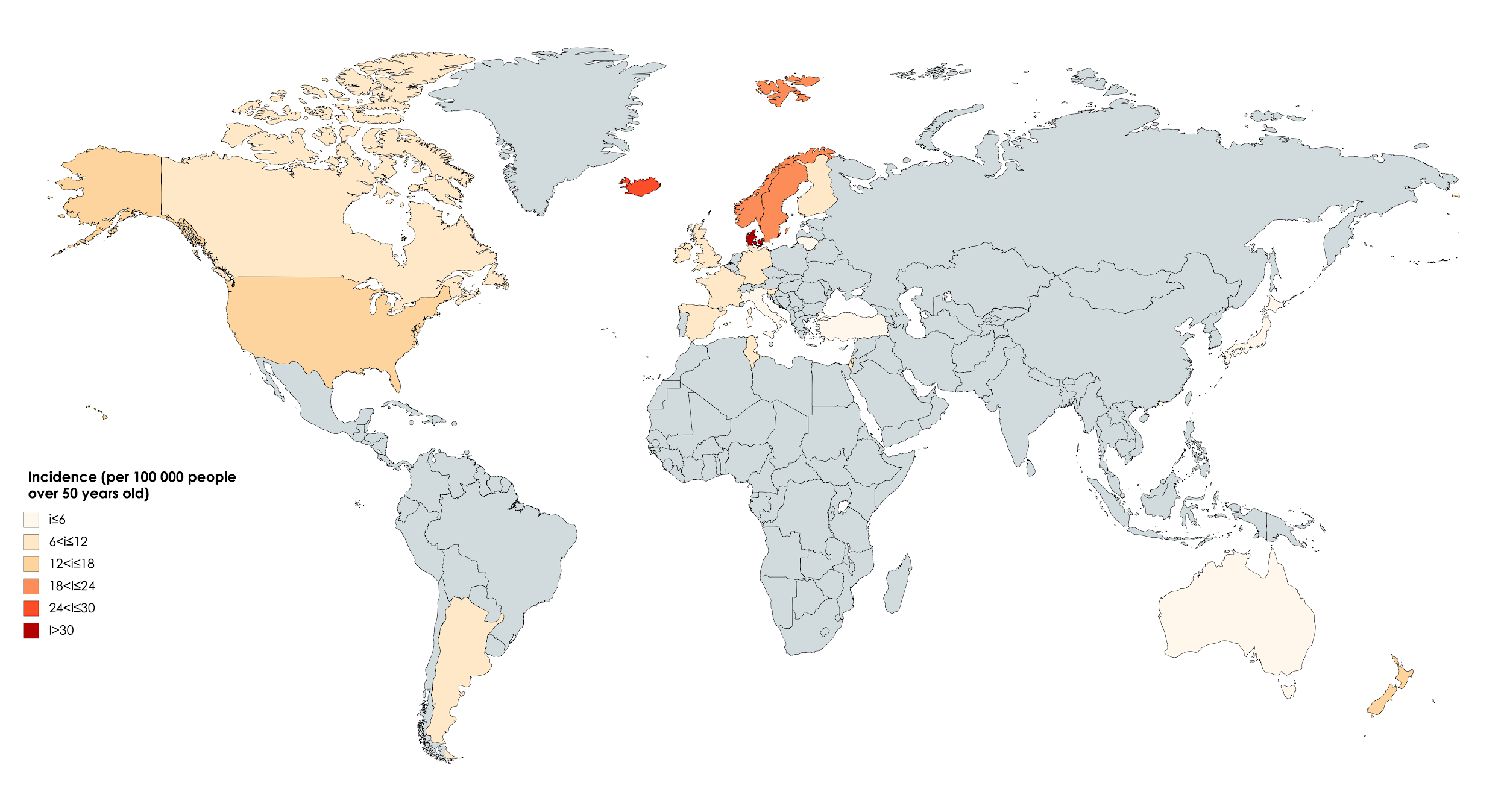


**Supplementary Figure 2 Global Incidence of Giant Cell Arteritis on the World Map**

***grey = no data**
